# Values as determinant of meaning among patients with psychiatric disorders in the perspective of recovery

**DOI:** 10.1038/srep27617

**Published:** 2016-06-08

**Authors:** Philippe Huguelet, Sébastien Guillaume, Sonia Vidal, Sylvia Mohr, Philippe Courtet, Lucile Villain, Chloé Girod, Roland Hasler, Paco Prada, Emilie Olié, Nader Perroud

**Affiliations:** 1 Department of Mental Health and Psychiatry, University Hospitals of Geneva, Geneva, Switzerland; 2 Department of Psychiatry, University of Geneva, Geneva, Switzerland; 3 Department of Emergency Psychiatry and Post-Acute Care, CHRU Montpellier, France; 4 Inserm, U1061, Université Montpellier, Montpellier, France; 5 Fondamental Foundation, Foundation of Scientific Cooperation, France; 6 Department of Emergency Psychiatry and Post-Acute Care, CHRU Montpellier, France

## Abstract

Recovery is a personal process of growth that involves hope, self-identity, meaning in life and responsibility. Determinants of *meaning* have not been explored among populations of patients with persistent psychiatric conditions. However, an evidence-based approach aiming at assessing such determinants should provide some insight into the psychotherapeutic aspects of recovery. We tested a model hypothesizing that some symptoms and social parameters of patients are related to *values*, and secondarily to meaning in life, and in turn that meaning is associated with various parameters, such as depressiveness and self-esteem. We assessed 176 patients with schizophrenia, anorexia, borderline personality disorder and bipolar disorder. Overall, our hypotheses proved correct: firstly, characteristics such as depression, hopelessness, self-esteem and the number of relationships influenced values; secondly, the presence and an enactment of values were associated with meaning, and thirdly, meaning was associated with some symptoms and social characteristics. This model was confirmed in the four psychiatric populations under study. These results support the relevance of addressing values and meaning in the recovery-oriented care of patients with persistent psychiatric disorders, in addition to other psychosocial interventions which are more systematically considered in this area.

In the context of a persistent mental disorder involving losses and functional disabilities, psychological recovery denotes the development of a fulfilling life and a positive sense of identity founded on hopefulness and self-determination^1^.

Up until now, psychotherapy work touching at the very core of the concept of recovery (the psychological dimension involving hope, meaning, identity and responsibility) has yet to be based on firm theoretical and empirical grounds. Psychotherapists have long developed concepts and approaches related to meaning; most of the time, these are based on philosophical literature and cases studies. Spinelli^2^ underlines that the “distinguishing characteristic of being human” is that we expect meaning from the world. Consequently, we are disturbed by lack or loss of meaning. The existentialism-oriented psychiatrist Yalom^3^ argues that anxiety in human life is related to issues such as *meaninglessness*. Corrie & Martin^4^ have described the relationships between existential and cognitive-behavioural therapy. While acknowledging that these two approaches use quite different stances and techniques, they propose that both rest on common concepts such as “meaning”: The search for meaning could therefore be the ultimate goal of both techniques.

Meaning involves at least two dimensions^3^: *Personal life-meaning* is concerned with one’s goals in life. This concept may be more impregnating for those who do not believe in a supra-entity. Camus^5^ and Sartre^6^ wrote about this quest for meaning, by emphasizing that human beings should accept responsibility for shaping their life-meaning (rather than by discovering a meaning from God or Nature). There can be *altruistic* personal life meanings (e.g. living for children, helping in an associative network, etc.) and *self-centred* meanings (e.g. succeeding in studies, fully experiencing the present moment…). Yalom^3^ describes secular activities that are likely to confer significance and purpose. Unsurprisingly, altruism, dedication to a cause and creativity are mentioned, but hedonism (in the sense of aiming at living fully in the moment) and self-actualization (i.e. self-development) are also included. *Cosmic meaning* concerns the spiritual dimension of our lives, i.e. the manner in which human life is integrated into the universe. Religions can provide some answers for those who believe in God, by providing comprehensive worldviews.

What are the determinants of meaning? Authors such as Yalom^3^ and Battista and Almond^7^ discussed or studied the conditions under which an individual develops meaning in life. Spirituality, self-esteem and close relationships may help to gain meaning in life. Debats *et al.*^8^, in a quantitative and qualitative study, associated meaningfulness to contact with self, others and the world. Battista and Almond^7^ stated that restoring self-esteem is a first step towards developing positive “life regard”. Beyond this necessary yet insufficient prerequisite, meaning would be correlated with phenomena further orientated towards life goals. In particular, meaning depends on the fit between the values, goals and needs of the individual and the values, goals and needs of the social structure in which he/she lives.

*Values* can be defined as “implicit or explicit principles orienting one’s action”. In terms of behavioural sciences, values are considered as reinforcing factors, the benefits of which are often delayed^9^. More specifically, in the context of Acceptance and Commitment Therapy (ACT), Wilson *et al.* define values as “…freely chosen, verbally constructed consequences of on-going, dynamic, evolving patterns of activity, which establish predominant reinforcers for that activity that are intrinsic in engagement in the valued behavioural pattern itself”. The capacity to establish values requires that humans are able to build psychological functions without the necessity of direct conditioning processes. Values are different from goals, which are achievable ends. Unlike goals, values cannot be completed or achieved in an absolute sense. For example, getting a degree is an achievable end. Education might be a value that can continue – and be modified - for a lifetime. Values can be determined according to domains such as work, family, education…^10^ or according to broader principles such as autonomy, power, altruism, and so on^11^.

Therefore, the postulate is that values foster a feeling of meaning^3^. For instance, if altruism is important to someone, organizing his/her life in accordance with this value may help fulfil a feeling of meaning. Conversely, living in a society where human rights are neglected can affect individuals for whom this value of altruism is important. This principle may be applied to patients with severe mental disorders, and whose values decline due to the psychological and social effects of their disorder.

## Meaninglessness and recovery

As mentioned above, one may infer that persons carrying the burden of a chronic psychiatric condition are challenged when searching for meaning. At the same time, Frankl^12^, a holocaust survivor, insisted that having a reason to live can help individuals make it through terrible ordeals, such as those he encountered in concentration camps. Despite the likelihood that existential therapy or ACT provides important tools in the pursuit of recovery, this topic has rarely and sparsely been addressed in literature. We therefore made the assumption that it is possible to conduct experimental research on this issue, by testing a formerly-hypothesized model^13^. This model is embedded in the bio- psycho**-** social paradigm^14^ and relies on the current principles of cognitive behavioural approaches^15^.

**Figure 1:**
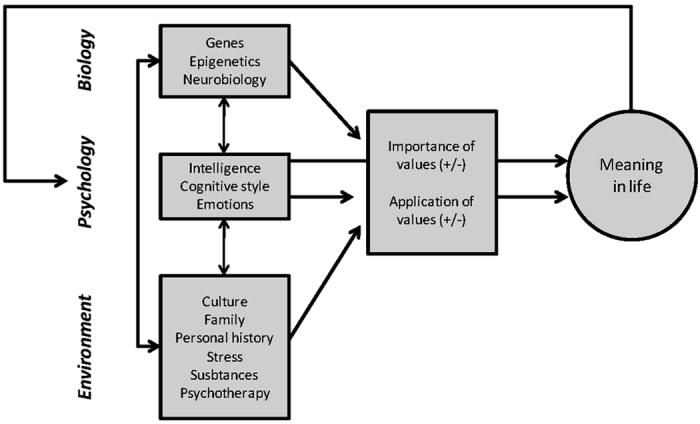
Meaning in life in the bio- psycho-social paradigm.

This model postulates that various biological (e.g. genetic), psychological (e.g. mood or cognitive “events”) and social (e.g. education or adversities) issues influence one’s values, first by allowing them to be present in one’s mind, and then by allowing them to be acted upon in current life. It follows that the fulfilment of values allows subjects to achieve a sense of meaning. Finally, the sense of meaning (or lack thereof) may retroactively bear an impact on some of the aforementioned bio-, psycho-, and social contexts of subjects, as discussed formerly by Debats^16^ (Fig. 1). For example, a subject suffering from depression may lose the opportunity to promote his/her values, due to negative thoughts and other symptoms related to his/her condition. This would alter his/her sense of meaning. This feeling of lack of meaning would in turn participate to an overrepresentation of negative thoughts related to depression, hence altering his/her well-being.

### Aims of the Study

In this research, we tested this model by hypothesizing that the psychological state of patients and some social parameters are related to values and secondarily to meaning in life. In turn, meaning is associated with various symptoms and social parameters. More specifically, according to our model, we went further in terms of *causalities*, by testing whether symptoms and social parameters influence values, which in turn influence meaning; retroactively, this specifically has an impact on symptoms and social parameters. We studied subjects with long term – yet stabilized - psychiatric conditions. Indeed the issue of meaning is likely to be challenged when life involves persisting symptoms and relapses with important social consequences. The study of four different psychiatric populations aims at providing a picture of distinct alterations of meaning and values depending on specificities of these disorders.

## Methods

### Subjects

Participants were adults who met the ICD-10^17^ criteria for schizophrenia (n = 67) or other chronic psychoses (schizoaffective disorders: n = 6 and other psychotic disorder: n = 2) (PSY), borderline personality disorders (BPD), anorexia nervosa (ANO), or bipolar disorder, type I (n = 18) or II (n = 17) (BD). They were randomly recruited from four psychiatric facilities: two public ambulatory facilities in Geneva, Switzerland, and two psychiatric units in Montpellier France. Among Geneva’s ambulatory facilities, one was specialized in the treatment of BPD, and the other treated patients with psychosis in an outpatient clinic or in assertive community treatment. The third and fourth psychiatric facilities were a hospital unit admitting patients suffering from eating disorders and an outpatient clinic assessing BD in Montpellier. PSY and BPD patients were stabilized, i.e., they had not been hospitalized for at least 6 months prior to the study. BD patients were assessed while being stabilized for several months and, as such, most of them did not suffer from current depressive episode (17 of them suffered from mild to moderate depressive episode according to the Beck Depressive Inventory (BDI-II) and the remaining 18 BD subjects had a score below 10 at the BDI-II suggesting that they did not suffer from a current depressive episode) and none of them from current manic/hypomanic episodes. ANO patients were recruited in an outpatient eating disorders (EDs) unit, in Montpellier, France. This is a second-line unit where patients are sent for EDs (or suspicion of EDs) for a multidisciplinary assessment, diagnostic confirmation, and organization of care both for outpatient and inpatient management. The mean Body Mass Index (BMI) for ANO was of 15.85 (SD = 2.12) indicative of a severe level of anorexia nervosa.

To avoid a selection bias related to the characteristics of subjects, patient lists were screened by the research investigators to identify patients who were eligible for the research, without *a priori*. The study was approved by the ethics committee of the University Hospital of Geneva and Montpellier’s ethics committee. Methods were carried out in “accordance” with the approved guidelines. All participants received detailed information about the study and gave their written informed consent.

### Assessment of symptomatology

Diagnostic information was obtained by systematically reviewing medical records and using the Mini-International Neuropsychiatric Interview (MINI)^18^ for PSY, BD and ANO patients and the Diagnostic Interview for Genetic Studies (DIGS)^19^ for BPD patients. The Positive And Negative Syndrome Scale (PANSS)^20^ was also administrated to PSY and BPD patients. We used the Beck Depressive Inventory (BDI II)^21^ in order to evaluate the current severity of depression and the Beck Hopelessness Scale (BHS)^22^ to assess the current level of hopelessness. In order to exclude current manic or hypomanic states in BD subjects, we used the Young Mania Rating Scale (YMRS)^23^, and the Altman Self-Rating Mania Scale (ASMS)^24^. A score below 12 at the YMRS and a score below 6 at the ASMS are indicative of a euthymic state. None of the BD subjects had a score above the thresholds for mania or hypomania on both scales (see Table 1).

### Assessment of values, meaning in life and other variables

The importance of values was established with the Valued Living Questionnaire^9^. The VLQ is a two part self-report questionnaire designed to identify the *importance* of 10 main domains in life (family relationship, intimate relationship, parenting, social relationship, professional life, education, hobbies, spirituality, citizenship and physical well-being) on a 10-point Likert scale. A second part measures the fit between the importance of each of the 10 domains and the subject’s actual commitments over the past week (*consistency*). The total score is a composite score, representing the degree to which patients live in accordance with the values they consider as important or not, with higher scores indicating higher importance and commitment.

In order to study meaning in life, we administered the revised version of the Life Regard Index (LRI-R)^25^. The LRI-R elicits the two underlying concepts of each personal meaning: the *framework scale*, measuring the degree to which the person can envision his/her life within some meaningful perspective and the *fulfilment scale* designed to assess the degree to which he/she sees himself/herself as fulfilling his/her life goals.

The participants’ self-esteem was established with the French translation of the Rosenberg’s Self-Esteem Scale (RSE)^26^, a 10-item scale measuring positive and negative feelings about oneself.

In order to obtain information about the subject’s relational context and interpersonal bonds, participants were asked about the most important or meaningful relationships of their lives. A composite variable was obtained by multiplying the importance of each person by the frequency of contacts per month. Religiousness and spirituality were investigated through a self-report questionnaire developed by Mohr *et al.*^27^ in order to determine the nature of religious and spiritual practices, as well as their frequency. Two variables were used: 1) The importance of religion/spirituality in daily life and 2) A variable reflecting both the importance given to religion/spirituality and the frequency of practices related to this importance. We obtained this variable by summing the scores for subjective importance and the scores for frequency of spiritual practices.

### Statistics

A one-way analysis of variance (ANOVA) was used to compare the clinical and demographic variables among the four clinical groups (ANO, BPD, BD and PSY). Linear regression with adjustment on age, gender and clinical groups was used to assess the association between VLQ total score and the following variables: BDI, BHS, self-esteem, number of relevant relationships, the frequency of contact with them, the importance of religion and spirituality in daily life and the frequency of practicing it. At this stage, as we tested the association between 5 independent variables (number of relevant relationships, the frequency of contact with them, the importance of religion and spirituality in daily life, the frequency of practicing it were considered as non-independent variables), and two different outcomes (LRI and VLQ), a correction for multiple testing was required. For a Bonferroni correction on the P values, we therefore used P = 0.05/[5 × 2] = 0.005 as a threshold for significance.

Variables significantly associated with VLQ total score were then used as independent variables in a multivariate linear regression model with adjustment on age, gender, and clinical category to assess their impact on values (VLQ total score). This method was used to follow the rule established by Peduzzi *et al.*^28^, according to which the variables included should not exceed the number in the smallest outcome group divided by 10. The same models were used to assess the association between LRI total score and these clinical and demographic variables. Multivariate linear regression models (2 models: one for VLQ and one for LRI) were used to reduce the number of tests performed and thus reduce the risk of type I errors. Thus only variables with P value < 0.05/2 ≤ 0.025 were considered as significant and subsequently used in the mediation analyses.

The variables that remained significant in the multivariate models (either with LRI total score or VLQ total score) were then used in a mediation analysis to test their effect on LRI total score through the mediating effect of VLQ total score. Mediation analyses were carried out using the “medeff” command, as implemented in STATA v.12.1. and described by Hicks and Tingley^29^ with 1000 simulations and 1000 bootstraps. Although resampling methods such as bootstrapping are effective in reducing type I errors and thus may partly account for multiple testing issues, we, in addition, considered only associations with P value ≤ 0.05/5 (5 mediation analyses) = 0.01 as significant. As several variables were not normally distributed, all analyses were standardized by means of z transformation ((score-mean)/SD). STATA SE v.12.0 was used for the analyses.

### Power calculation

The sample of 176 subjects had 99% and 95% power to detect an association with LRI and VLQ at an alpha level of 0.05 and 0.005 respectively. The power dropped to 65% when considering the subsamples of ANO, BPD, and BD for an alpha level of 0.05 and to 92% for the subsample of PSY subjects. Our study was thus enough powered to detect even small associations in the whole sample and associations of mild to moderate magnitude when considering the subsamples.

## Results

### Clinical and Demographic characteristics of samples. 

The four samples significantly differed on almost all the clinical and demographic variables, the only exception being measures of hopelessness. In addition, BD type I subjects were more often women (n = 14; 78%) than BD type II subjects (n = 4; 24%) (Table 1).

**Figure 2:**
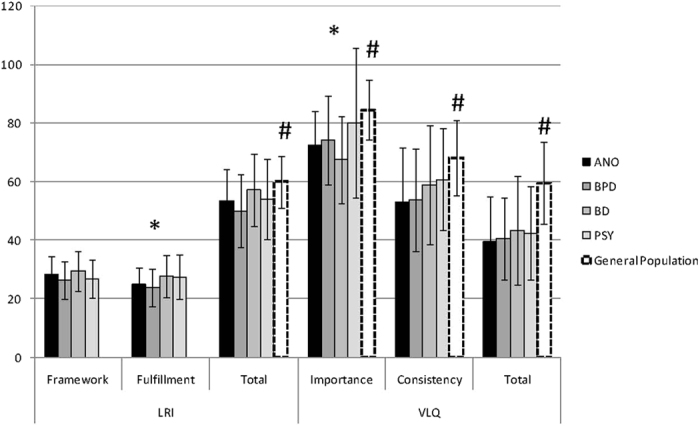
VLQ and LRI total scores and subscales in the four clinical groups: chronic psychoses (PSY), borderline personality disorders (BPD), anorexia nervosa (ANO), and bipolar disorders (BD). Error bars = standard deviation. LRI total score [60.02 (SD 8.92)] and VLQ total and subscale scores for the general population are respectively taken from Debats *et al.*^17^ and Wilson *et al.*^9^. *Significant (p < 0.05) between-group difference considering the four clinical groups. ^#^Significant (p < 0.05) difference between scores found in the general population and scores in the whole clinical sample.

Although samples were similar in the LRI total score, they differed in one of the subscales (LRI Fulfilment (F = 2.93(3/175), p = 0.035), with BPD having the lowest scores and BD the highest ones. Similarly for the VLQ, there was no difference between groups when considering the total score, but differences emerged when looking at the importance of values in daily life (F = 3.56(3/175), p = 0.015) with BD subjects having the lowest scores and PSY having the highest ones (Fig. 2). Considering BD type I and type II subjects, none of the clinical variables differed between groups. Similarly there was no difference for the VLQ and LRI scores between BD type I and type II subjects.

### Values and meaning of life and their associations with clinical and demographic variables

In the entire sample, after adjustment on age, gender and main psychopathology (ANO, BDL, BD or PSY), there was a significant association between values (and the possibility to use them in daily life), as measured by the VLQ, and meaning in life (β = 0.49; p < 0.0001). This association was significant in each of the clinical samples taken individually.

Values were significantly associated with severity of depression (β = −0.41; p < 0.0001), hopelessness (β = −0.47; p < 0.0001), measure of self-esteem (β = 0.40; p < 0.0001), the number of relevant relationships (β = 0.35; p = 0.0001) and the frequency of contact with them (β = 0.30; p = 0.0005), the importance of religion and spirituality in daily life (β = 0.27; p = 0.002) and the frequency of practicing it (β = 0.20; p = 0.018).

After adjustment on age, gender and category of psychopathology, the following variables remained significantly associated with the VLQ total score: hopelessness (β = −0.38; p = 0.005), the number of important relationships (β = 0.23; p = 0.006), and the importance of religion/spirituality in daily life (β = 0.16; p = 0.037).

Meaning in life (LRI total score) was significantly associated with severity of depression (β = −0.62; p < 0.0001), hopelessness (β = −0.68; p < 0.0001), self-esteem (β = 0.60; p < 0.0001), the number of relevant relationships (β = 0.34; p = 0.0001) and the frequency of contact with them (β = 0.18; p = 0.037), the importance of religion and spirituality in daily life (β = 0.28; p = 0.001) and the frequency of practicing it (β = 0.25; p = 0.003).

In a multivariate analysis that includes all the variables associated with LRI, and after adjustment on age, gender and category of psychopathology, only the following variables remained significantly associated with the LRI total score: severity of depression (β = −0.17; p = 0.018), hopelessness (β = −0.40; p < 0.0001), self-esteem (β = 0.16; p = 0.018), and the number of important relationships (β = 0.13; p = 0.017).

### Mediation analyses

**Figure 3:**
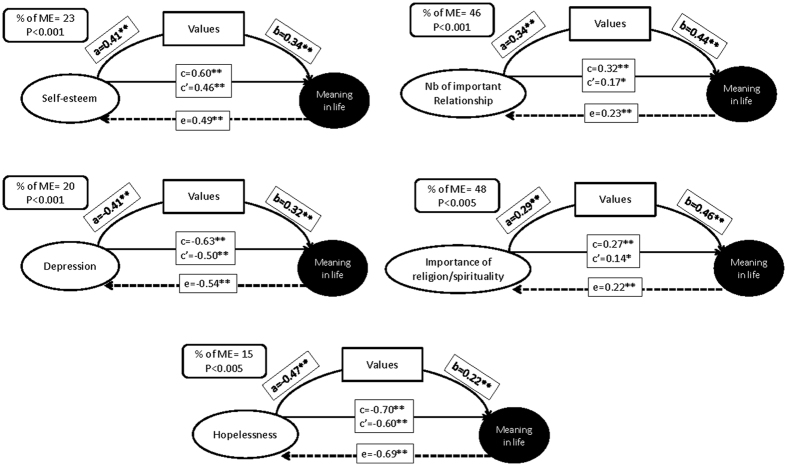
Standardized path coefficients of the mediation model (mediator = values) of valence of self-esteem, severity of depression, hopelessness, number of important relationships and importance of religion/spirituality in daily life on meaning in life. c = total effect of independent variables on the outcome (ab+c’); c’ = direct effect of independent variables on the outcome; a = effect of the independent variable on the mediator; b = effect of the mediator on the dependant variable. E = direct effect of LRI on the following dependant variables: self-esteem, severity of depression, hopelessness, number of important relationships and importance of religion/spirituality in daily life on meaning in life. **p < 0.001, *p < 0.05.

The mediation analyses were performed on variables significantly associated either with the VLQ total score or the LRI total score in multivariate models. After adjustment on age, gender and category of psychopathology, values significantly mediated the association between depression severity (p < 0.001), hopelessness (p < 0.005), self-esteem (p < 0.001), the number of important relationships (p < 0.001), the importance of religion/spirituality in daily life (p < 0.005) and meaning in life (Fig. 3). These models showed the same direction and the same magnitude of effect when considering each category of diagnosis individually.

Inversely, namely by testing whether values would mediate the effect of meaning in life on the clinical and demographic variables, only the models comprising the number of important relationships (p < 0.05) and the importance of religion/spirituality in daily life (p < 0.05) were significant.

## Discussion

The present study focused on values as a mediator between various symptoms or social dimensions and meaning in life. Furthermore, we tried to assess whether, *in turn*, meaning in life impacts these parameters. These hypotheses, stemming largely from existential literature and clinical intuition, were confirmed by our analyses, for four different psychiatric conditions, all of them being characterized by a chronic course and a high level of severity.

### Meaning and values

LRI results, as indicators of meaning in life, were used in other populations and it is interesting to compare these results with those from our sample. Debats *et al.*^30^ found a total score of 60.02 (SD 8.92) for a sample taken from the general population. This is significantly (p < 0.0001) higher than the score found in our clinical sample (four groups taken together: 53.73 (SD = 12.88)), which suggests that suffering from a chronic and disabling condition is associated with reduced meaning in life.

Concerning the effect of diagnoses on LRI results, differences were found on LRI Fulfilment, with BPD having the lowest scores and BD the highest ones. This may reflect the fact that BPD patients have preserved a framework of meaning in life, but are unable to implement it in their life. BPD has recently been thought to be the result of deficient mentalizing capacities, i.e. the capacity someone has to consider the mental states that underlie our actions and those of others. This poor mentalizing process might impact the ability a subject has to fulfil life goals, despite the fact that these goals are clearly well defined in the mind of the subject. Further research is clearly needed in this area in order to better understand the reasons why BPD subjects show deficits in the fulfilment of goals related to meaning in life.

Values in the general population were measured with the VLQ by Wilson *et al.*^9^, who found a mean score of 84.65 (SD 10.38) for “Importance”, 68.11 (SD 12.82) for “Consistency” and 59.52 (SD 14.14) for the Valued Living composite or total score. These results are clearly and significantly (p < 0.0001) greater than the scores found in our entire sample, which were as follows: 75.16 (SD = 20.31), 57.7 (SD = 18.48), and 41.70 (SD = 15.99) respectively. These results are compatible with the conditions of our patients’ sample. It makes good sense that people featuring symptoms such as depressive ideation and/or social adversities show impaired values.

Comparing VLQ values across diagnoses revealed differences for “importance” of values in daily life, BD subjects having the lowest scores and PSY having the highest ones. This should reflect varying coping styles depending on diagnoses, PSY patients giving more importance to values, while having only limited opportunities to implement them in their life. To note, the results for the PSY group could reflect (at least in part) the possible influence of some alterations of cognitive function. Indeed, even if the vast majority of patients with psychosis do not feature massive cognitive decline over time, their relative “lack” of meaninglessness and alterations in values’ score could be related to some “mild” neurocognitive alteration preventing them from a clear view of their social condition.

### Symptoms, Values and meaning: General model

In the present study, for patients who suffer from severe mental disorders, the association between symptoms and meaning was mediated by values, preferentially in the direction “symptoms = > values = > meaning”. This suggests that symptoms such as depression, hopelessness or psychotic symptoms alter values. In turn, a lack of values and/or the implementation of values in one’s life alter meaning. The mediation analyses showed that the models tested were the most significant pathways.

The loop involving values and meaning was found for multiple variables. It appears to be quite logical to find that this process involves depression, hopelessness and low self-esteem. The fact that a number of important relationships are also involved could confirm existentialists’ claim that meaning is fostered by human bonds^3^,^8^,^31^. Religion/spirituality may also provide values and meaning^32^. Data confirms such an association.

If some symptoms impact meaning, what is there to say about a possible reverse impact of meaning (or rather meaninglessness) on symptoms? Overall, some studies^16^,^30^,^33^,^34^, suggested that meaning in life plays a role in one’s state and reciprocally. Our data goes further by examining the influence of meaning on numerous characteristics or symptoms, in four different psychiatric disorders characterized by a chronic course.

Existentialists^3^ gave case reports describing how lack of meaning affects patients in clinical settings (“Meaning as a question of life or death”). For patients suffering from psychosis, one may postulate that meaning can be altered, as argued by Berna *et al.*^35^. These authors showed in their research that meaning-making was impaired for self-defining memories among patients with schizophrenia. Beyond this finding, we may postulate that this limitation for giving meaning retrospectively may also be present when trying to give meaning to present or future events.

In research more specifically oriented towards the relation between meaning and clinical state, Debat *et al.*^30^ showed a strong negative association between LRI scores and depression, anxiety, interpersonal sensitivity and general psychopathological distress. At that time, the authors admitted that it was not possible for them to conclude whether lack of meaning in life was the cause or the effect of psychological problems. More recently, Ju *et al.*^33^ studied older adults with the hypothesis that well-being was partially mediated by meaning in life. Among these healthy subjects, the authors showed that optimism was indeed associated with well-being, while meaning in life partially mediated this association. It was therefore suggested that optimistic attitudes help perceive meaning in life, which in turn contributes to better subjective well-being. In another study, carried out among a population of students, presence of meaning in life was associated with decreased suicidal ideation^34^. These authors also showed that meaning in life mediated the relationship between “perceived burdensomeness” and “thwarted belongingness” vs. suicidal ideation.

### Limits

Subjects’ clinical and socio-demographic characteristics were quite heterogeneous across the diagnostic groups, partly due to the different diagnoses themselves, but also because of the recruitment procedure, with some subjects being hospitalized and others not. Although this aspect limits comparisons, it should also improve the generalization of the data, as the associations between subjects’ characteristics, values and meaning was found for all groups. Also, as mentioned in discussion, cognitive function, particularly for the PSY group, may partially account for some higher scores on values and meaning. Further research should assess the role of cognitive function on these variables. Patients in the different group were not free from comorbidities such as depressive or anxiety disorders for BPD for instance. Although these comobidities were assessed using the MINI or the DIGS, for power issues and in order to avoid ending up with small groups not allowing proper comparisons between them, we decided to stick to the main diagnosis for which the patient was referred to one of the facilities.

The choice of values as a mediator between symptoms and meaning merits discussion, as some authors found other domains “directly” related to meaning (e.g. spirituality, self-esteem, interpersonal bond), although they failed to assess values. We decided to formulate our main hypothesis on the definition of values by Wilson’s *et al.*^9^, which, partially at least, encompasses some of these issues. There are therefore some tautological aspects to our mediating analyses that may account for the significant findings.

Furthermore, detailing items on the scales used for symptoms, values, and meaning shows that some of these items may be, to some extent, similar across constructs^36^. For instance, the LRI features an item, “I really feel good about my life”, which is likely to invoke a construct such as mood; this construct is reflected by the BDI^21^ evaluating the severity of depression. Beyond these limits, which may lead to consider our results as somewhat tautological, we should keep in mind that this type of research on cognitive processes involves delimitation of constructs which are *a priori* and to some extent arbitrarily delineated and inevitably overlapping.

However, keeping in mind these important limitations, the fact that our statistics repeatedly show an association between symptoms and meaning, with values as a mediator, are consistent with the appraisal of these patients with mental disorders in a clinical perspective.

### Clinical implications

This gives some weight to the idea that an intervention on the issues of meaning would be pertinent among a psychiatric population, in particular, by considering that meaning relies on values and that meaninglessness may alter well-being and foster symptoms. Overall, one may underline that clinicians should help patients find solutions to their current problems, sometimes with concrete issues, while *at the same time* retaining a capacity to mentalize what it means to him/her in terms of lifetime trajectory^16^. In other words, the idea is to consider whether any project is meaningful for a patient.

Some authors emphasize the fact that clinicians should help patients recover well-being from aversive events. Yet, ultimately, gaining an increased sense of life’s meaning would entail a more positive outcome^36^. In this perspective, Lapierre *et al.*^37^ found that an intervention targeting meaning in life (with a personal goal intervention program) was quite effective to reduce suicidal ideation.

The literature on therapy itself, while less grounded on research data, also gives some important insights. This pertains either to existentialism or to cognitive-behavioural therapy. Besides, Corrie & Martin^4^ claimed that meaning could be a central framework that unites both existential and cognitive-behavioural techniques. Some insights are given on these approaches in the perspective of the data from this study.

Existential approaches have considered how to define and address meaning in therapy^3^,^31^. Nevertheless, further work is needed for building a specifically recovery-oriented care addressing meaning^37^. Van Deurzen^31^ claims that only existential therapy can address all dimensions of meaning. Yalom^3^ suggests that considering values may be an easier starting point when talking to patients who are not immediately comfortable with considering the lack of meaning. Assistance in finding a meaning should be provided to patients in the context of their psychiatric disorder, which often prevents them from fulfilling their projects. Yalom also emphasizes that working on life meaning is likely to involve other “existential” issues such as death and responsibility. Overall, existentialists state that commitment may increase the possibility for building a coherent life scheme^5^. Clinicians should work on the obstacles that prevent the patient from achieving this goal.

The particular issue of recovery is a domain to take into account when treating patients facing long-term mental disorders with alteration of various domains in life. As Lieberman wrote recently^38^, “Mental illness is a medical condition – but it’s also an existential condition”. This issue is particularly important when working with a recovery-based approach, as some clinicians may neglect its psychological dimension by limiting their action to a transformation of systems^39^ and/or to psychosocial rehabilitation that is behaviourally grounded^40^. In this perspective, as described in our introduction, considering meaning is an important treatment goal. Based mostly on the cognitive-behavioural tradition, this can be done by considering the so-called “3^rd^ wave” acceptance and commitment therapy (ACT)^10^. ACT aims at achieving balance between acceptance (of what can’t be changed) and commitment (i.e. engaging in committed actions). This is obviously the core of what can be done in a recovery-oriented approach: finding meaning in new goals beyond the effects of unresolved symptoms and possible impairments. ACT also involves considering values as an important issue^10^. Therapists should indeed help patients overcome obstacles, hence allowing them to move actively in the direction of chosen values. This may be more efficient than considering meaning directly, as Yalom also stated. Our data provides some support to this approach: values appear to be associated with meaning.

Values therefore appear both as a motivational agent and a path to meaning. Meaning itself could be both a goal and an issue altering well-being and symptoms. Faced with these intricate, albeit important issues, further research is needed. In the meantime, as stated some 20 years ago^16^, clinicians should consider that meaningfulness and meaninglessness are more than mere philosophical concepts.

In conclusions, we found that values of patients suffering from chronic psychiatric disorders significantly mediated the association between their symptoms (hopelessness, depression, and self-esteem), social parameters (number of important relationships, and importance of religion/spirituality) and their meaning in life. These results support the relevance of addressing values and meaning in the care of patients with persistent psychiatric disorders.

## Additional Information

**How to cite this article**: Huguelet, P. *et al.* Values as determinant of meaning among patients with psychiatric disorders in the perspective of recovery. *Sci. Rep.* **6**, 27617; doi: 10.1038/srep27617 (2016).

## Figures and Tables

**Table 1:** Clinical and Demographic characteristics of the four clinical samples.

		Anorexia nervosa(N = 28)		Borderline Personality Disorder(N = 38)		Bipolar Disorder I or II(N = 35)		Psychotic Disorders(N = 75)			
	*Mean*	*SD*	*Mean*	*SD*	*Mean*	*SD*	*Mean*	*SD*	*F(df)*	*p*	
Age		25.46	6.74	33.83	9.97	43.56	11.72	45	11.45	28.29(3/175)	<0.0001
Years of schooling	14.64	4.24	14.74	3.99	16.17	3.7	12.55	3.71	8.04(3/175)	<0.0001	
PANSS	Pos. Symptoms	–	–	10.21	3.49	–	–	15.24	7.24	14.56(1/107)	0.0002
	Neg. Symptoms	–	–	9.21	1.88	–	–	19.72	8.66	54.37(1/107)	<0.0001
	Psychopathology	–	–	28.87	7.41	–	–	35.94	12.11	8.92(1/107)	0.0035
	Aggression	–	–	7.45	3.27	–	–	6.42	4.07	1.82(1/107)	0.181
BDI		25.79	10.84	23.39	11.64	13.7	9	17.04	10.62	9.77(3/174)	<0.0001
BHS		8.11	5.46	9.23	5.16	7.7	4.16	7.64	4.8	0.98(3/174)	0.402
YMRS		–	–	–	–	2.43	4.1	–	–	–	–
ASMS		–	–	–	–	2.76	3.96	–	–	–	–
Self–esteem		22.61	5.77	22.87	6.92	28.37	6.27	29.01	5.99	13.13(3/175)	<0.0001
Number of important relationships		3.89	0.99	4.14	1.13	4.17	1.1	2.61	1.19	24.35(3/174)	<0.0001
Number of times an important relation was seen/months		191.43	135.5	257.9	151.03	245.96	168.13	87.06	105.54	18.67(3/174)	<0.0001
Importance of religion/spirituality in daily life		3.89	3.49	4.7	5.27	4.03	4.25	9.41	5.44	15.99(3/174)	<0.0001
Spirituality (freq + importance)		4.79	4.5	5.24	5.91	5.26	5.72	10.09	5.7	11.39(3/174)	<0.0001
LRI	Framework	28.32	6.02	26.14	6.45	29.43	6.72	26.75	6.65	2.03(3/175)	0.112
	Fulfilment	24.96	5.67	23.89	6.5	27.73	7.09	27.39	7.59	2.93(3/175)	0.035
	Total	53.29	10.94	50.04	12.56	57.16	12.26	54.17	13.71	1.94(3/175)	0.125
VLQ	Importance	72.53	11.56	74.16	15.18	67.45	14.79	80.21	25.5	3.56(3/175)	0.015
	Consistency	52.92	18.83	53.81	17.62	58.97	20.33	60.83	17.51	1.97(3/175)	0.121
	Total	39.43	15.44	40.46	13.96	43.37	18.56	42.32	16.03	0.41(3/175)	0.746
		*N*	*%*	*N*	*%*	*N*	*%*	*N*	*%*	*X2(df)*	*p*
Gender (Female)		27	96.43	35	92.11	18	51.43	30	40	45.99 (3)	<0.0001
Marital status (in couple/married)		4	14.29	5	12.5	17	47.22	6	8	27.13 (3)	<0.0001
Number of children	0	27	96.43	28	70	14	38.89	58	77.33	29.17 (6)	<0.0001
	1	1	3.57	7	17.5	10	27.78	8	10.67		
	2 or more	0	0	5	12.5	12	33.33	9	12		
Working or in school		17	60.71	14	35	16	44.44	4	5.33	39.34(3)	<0.0001
Number of hospitalizations	0	1	3.57	14	35.9	8	22.86	1	1.35	49.04(9)	<0.0001
	1	12	42.86	10	25.64	8	22.86	9	12.16		
	2	2	7.14	5	12.82	2	5.71	9	12.16		
	3 or more	13	46.43	10	25.64	17	48.57	55	74.32		

PANSS: Positive And Negative Syndrome Scale, BDI: Beck Depressive Inventory, BHS: Beck Hopelessness Scale, LRI: Life Regard Index, VLQ: Valued Living Questionnaire, YMRS: Young Mania Rating Scale, ASMS: The Altman Self-Rating Mania Scale.
